# Knowledge, attitude, practices and their associated factors towards diabetes mellitus among non diabetes community members of Bale Zone administrative towns, South East Ethiopia. A cross-sectional study

**DOI:** 10.1371/journal.pone.0170040

**Published:** 2017-02-02

**Authors:** Chanyalew Worku Kassahun, Alemayehu Gonie Mekonen

**Affiliations:** Department of Nursing, College of Medicine and Health Sciences, Madda Walabu University, Bale Goba, Ethiopia; National Institute of Health, ITALY

## Abstract

**Background:**

Diabetes kills more than 4.9 million adults per year. It becomes rapidly increasing, non-communicable disease—a major threat to global public health particularly in Sub-Saharan Africa. Though previous studies among diabetic patients were focused in health institution, limited knowledge, attitude and practice were seen. There is no study conducted about diabetes related to knowledge, attitudes, practice and associated factors in the community level.

**Objective of the study:**

The study assessed knowledge, attitude, practices, and its associated factors towards diabetes mellitus among non diabetic community members of Bale Zone, Ethiopia.

**Methods:**

Community based cross-sectional study was conducted from November 15 to December 15, 2015 among 605 non diabetic community members of Bale Zone administrative towns. Data was collected using pretested structured face-to-face interview after taking informed written consent. Respondents were selected by systematic random sampling. The data was entered into EPI data version 3.1 and analyzed using Statistical package for social sciences version 20. Odds ratio and 95% confidence interval were calculated and P<0.05 was considered statistically significant. Finally, multivariable logistic regression analysis was performed to indicate the independent predictors of knowledge, attitude and practice.

**Result:**

Response rate of the study was 98.2%. About 52.5% of participants were knowledgeable, 55.9% and 56.6% had good attitude and practice respectively. Earning average monthly family income of ≤500 Ethiopian birr (AOR = 0.4, CI = 0.2, 0.6) and 501–1000 (AOR = 0.4, CI = 0.2, 0.7), heard about diabetes (AOR = 4.4, CI = 1.9, 10.2), had diabetes health education exposure (AOR = 5, CI = 2.5, 9.7) resulted to have good diabetes knowledge. Student, (AOR = 5.1, CI = 2.1,12), government/private employee (AOR = 3,CI = 1.4,6.7), merchant (AOR = 2,CI = 1.1,3.6) and Knowledgeable (AOR = 3, CI = 2.1, 4.7) subjects had positive attitude towards diabetes. Having college and above educational level (AOR = 0.33, CI = 0.16, 0.7), having good attitude towards diabetes (AOR = 2, CI = 1.3, 3) had good practiced.

**Conclusion:**

Considerable limited knowledge, attitude and practices were seen. A great emphasis on health education regarding symptoms and risk factors modification for diabetes are necessary.

## Introduction of the study

Diabetes mellitus (DM) is a disturbance in the metabolism of carbohydrate, fat, and protein that is caused by due to lose of insulin producing cells in the pancreas or decreased tissues sensitivity to insulin that results in increased level of glucose in the blood[1,2].

Following life-style changes, global prevalence of DM is increasing rapidly providing a worrying indication and major threat to global health. This consumes the nation’s health care budget. Unless interventions are created through community awareness; Dm is predicted to be the world’s main disablers and killers of the working age groups in the next 20 years [1, 3]. It affects the socio economic status of all income level countries in both urban and rural populations. It affects. The socio economic effect is worst in the poorest countries [1].

In 2014 the International Diabetes Federation’s (IDF’s) reported that 387 million and 22 million adults had diabetes worldwide and in Africa respectively. This consumed 11% of worldwide health care budget; the number will increase more than 592 million in 2035. Africa will account the highest growth (42 million) with 4.9 million deaths per year. Majority are progressing towards complications without awareness. Moreover, around 80% of the total numbers affected are living in low- and middle-income countries [3,4].

Though it was once considered a rare disease in sub-Saharan African; more than 12 million people have diabetes and 330,000 diabetes-related deaths and projected that sub-Saharan Africa will have the highest growth of any region in the world where less than 1% of health expenditure is allocated for diabetes. In Ethiopia, diabetes cases are estimated 4.84%, of which 1.6 million undiagnosed, 34,263 diabetic related deaths that will cost more than 33 billion dollar annually. The prevalence is almost equal between male and female[4–8].

Diabetes increased risks of dying with cardiovascular diseases (primarily heart diseases and strokes), chance of limb amputation, kidney failure [9]and responsible for 4.8% cases of blindness worldwide [10]. It exerts a negative pressure in the control of infectious diseases like tuberculosis and HIV. Moreover, DM affected more than 21 million live births of pregnancy [11].

A study report in a semi–urban populations of Omani showed that the study subjects’ knowledge level regarding diabetes definition, symptoms, and complications were 46.5%, 57.0%, and 55.1%, respectively [12]. Around 33.75% knew the definition of DM and 45% described DM as a chronic disease in a study conducted among Students at Al-Balqa' Applied University[13]. Study in Mongolia population showed about 50% of Sub population and 1/5 of the total population had never heard the term DM, high level blood sugar symptom misunderstanding[14]. Another study in rural Bangladesh showed that 93% heard DM, 50% reported physical inactivity is a risk factor and dietary modification control DM by 40% -69%[15,16].

Around 58.1% had poor knowledge score among rural adult community in Malaysia[17] and awareness were 35% in Mangalore Medical College Students [18], while 43% had awareness of DM in Tarlai (rural Islamabad)[19]. Study among rural populations in India, showed 49.9% had knowledge, and most of them had right attitude[20]. In addition, In a comparative study in rural Indian state showed 28.2%, 36.1% and 40.3% diabetes patients had correct knowledge, attitude and practice, but lower score in non-diabetic groups [21]. In another study in Waghodia, India showed that 17.6% of participants had good attitude, 35% responded positively on uses of planned diets, 84% were checking their blood glucose level, and 74% performed regular exercise [22].

In a study in urban and semiurban population of Peshawar, Pakistan showed knowledge of symptoms and complications were 47.1% and 30.8%, Excessive sugar intake, obesity, family history, lack of physical activities and stress were acknowledged by 46.2%, 42.3%, 39.3%, 33.4%, and 31.8% of the subjects respectively [23]. In a qualitative study in Cameroon showed that most heard about DM, but about third of them had limited knowledge on risk factors and its treatments [24].

A study in a rural population of Sudan showed 15% had adequate knowledge, identified genetics (57.2%) and nutritional habits (46.9%) as risk factors, and retinopathy (31.1%) and cardio vascular diseases (16%) as complication[25].In Kenya 27% of the respondents had good knowledge on diabetes, 75% had poor dietary practices and 72% did not participate in regular exercise, and over 80% did not monitor their body weight, good knowledge had association with good practices[26]. In Debre Tabor, Ethiopia, 49% and 39.5% had good knowledge and good attitude towards diabetes mellitus respectively; in addition, positive relations between knowledge and positive attitude were seen[27].

In a cross sectional study in Bangkok and other central provinces of Thailand showed educational and age level brought diabetes knowledge differences [28]. While in South-Eastern Nigeria showed that age above 50 years, being female, married, earning more, attended secondary education, visiting health facilities had good diabetes prevention practice [29].However; female gender was an independent determinant of low general knowledge about diabetes in a cross sectional study in Zimbabwe [30]and men were more knowledgeable than women in study on the self-care knowledge on diabetes among diabetic patients in India [31,32].But dietary self-care was difficult in men than in women in Iran[33].

Gender, age, educational level, socio economic statues, family history of DM, obesity, exercise and smoking habit were factors of knowledge in a study conducted in Sudan, Kingdom of Saudi Arabia, Bangladesh, Islamabad and Ethiopia [15,19,27,34]. In Kenya, there was a direct relationship between levels of education and good knowledge on diabetes; but not across genders. In addition, participants with good knowledge of diabetes had good practices[35].

In a study conducted to Bahawalpur, Pakistan showed living in the urban, higher socioeconomic status, female gender had higher awareness, but lack of awareness were observed among illiterate and poor and rural populations[36].

Community’s knowledge can help to assess causes, risk of diabetes and motivate them to seek proper treatment and care. Although some studies in Africa or elsewhere, they focused on knowledge, attitude and practice of diabetic patients. Even studies in Ethiopia relied on its prevalence at community level and self-care knowledge at health care setting level. Therefore, this study assessed Knowledge, attitude, practice, and its associated factors towards diabetes mellitus among community members of bale zone administrative towns, South East Ethiopia, 2015.

### Operational definition

- **Attitude:** The way a community thinks and behaves toward DM. It is measured by 11 questions with five point Likert’s scale. All individual answers to attitudinal questions were computed to obtain total scores; then, mean score was calculated to categorize as having good attitude (if participants scored ≥ mean score)or poor attitude(if Participants scored < mean score).
- **Knowledge:** It is the awareness of the community about diabetes mellitus. It is measured by calculating the mean score of the 31 items and categorized as knowledgeable (if participants scored ≥ mean score of the correctly answered questions)or not knowledgeable (if participants scored <mean score of the correctly answered questions).
- **Practice:** The habitual community involvement to prevent DM. It is measured by 5 questions with five point Likert’s scale. All individual answers to practice questions were computed to obtain total mean scores and categorized as good practice (if participants scored ≥ mean score)or poor practice (if participants scored < mean score).

## Methods and subjects

Community based cross sectional study was conducted among selected non diabetic community members of Bale Zone Administrative towns, Ethiopia from November 15 to December 15, 2015. The Zone has three administration towns (Robe, Goba and Ginnir) and are located from 435–605 km far from Addis Ababa, the capital city of Ethiopia[37].Respondents were eligible if their age were≥18 years, and not too ill to be interviewed, willing to participate and were available during the data collection period; and have lived at least six months in Bale Zone Administrative towns. Participants were excluded if they were homeless and have clinically proven DM.

A total of 605 study participants were selected using single proportion formula by assuming Z α/2 = 1.96 (standard score value for 95% confidence level of two sides normal distribution), p = 49% (Good DM knowledge of community investigated at Debre Tabor town, Ethiopia[27], d (tolerated margin of error) = 5%, Non response rate = 5% and Design effect = 1.5.

After getting the number of households from zonal office, the calculated sample size was allocated proportional to the size of population in each administrative town. Then, kebeles from each administrative town were selected using simple random sampling and households from each selected kebeles were chosen using systematic random sampling every 19 households. Ten data collectors and two supervisors collected the data by moving from house to house after they took one-day training. The first household was chosen by lottery method, and systematic sampling technique was used in the subsequent households. The first person to be encountered in the household meeting the age criteria was interviewed. For those who failed, a second person was interviewed and if more than one individual meeting the age criteria were present in the same household, lottery method was used. In their absence, the next household was searched. For the absentees, arrangements were made for their follow-up interview and a maximum of three attempts were made to contact every eligible member during the study period.

The dependent variables were Knowledge, Attitude and Practice levels while the independent variables were demographic information (age, sex, marital status, and level of education, occupation and average family monthly income, and family history of DM), previous awareness about DM, exposure to health education and type of sources of information.

Structured interviewer administered questionnaire was designed by the researchers after reviewing literatures. The first part of the questionnaire covered the demographic information that included age, sex, and marital status, level of education, occupation, and average family monthly income, and family history of DM, exposure to health education about DM and health information medias like televisions or radios. The second part assessed general knowledge about diabetes. What is DM about, risk factors of DM, signs and symptoms of DM, Control and management DM and complications of DM? Respondents answered either “Yes’ or “No” or “Do not know”. The third part assessed the attitude of the respondents towards DM; and the final part covered the community’s’ practice to prevent DM. Five point Likert scale was used to assess attitude (5 = Strongly Agree, 4 = agree, 3 = neutral, 2 = disagree and 1 = strongly disagree) and practice (5 = very frequently, 4 = frequent, 3 = not sure,2 = less frequent and 1 = not at all) related to DM. Questionnaire was pretested with 5% of the total sample size in Dodola town, on 5% of the actual sample size outside of the study area in Dodola town, which is an urban district next to the study area two weeks before actual data collection. Pretest was used to assess the suitability of the content, clarity, sequence and flow of the questionnaire. The questionnaire was then being refined for final use.

All questionnaires were prepared in English language and then translated to Afan Oromo and Amharic (local language) which were used for data collection and re-translated back to English to check for any inconsistencies.

Mean scores of knowledge, attitude and practice about diabetes mellitus were calculated. To calculate the mean score of knowledge participants answered “Yes” was considered as correctly answered and those who answered “No” and “I do not know” were considered as not answered correctly. Therefore, knowledge mean was calculated from the correctly answered knowledge question. As result, the mean score was used to classify the knowledge level of the respondents in to two groups (knowledgeable and not knowledgeable). Respondents who scored mean (14.83) and above the mean score of the correctly answered questions were classified as knowledgeable, less than mean score of correct answers was classified as not knowledgeable. Likert’s scale was applied to measure the attitude and practice level. All individual answers to attitudinal and practice questions was computed to obtain total scores and calculated for means (1.56 for attitudinal and 1.57 for practice). The mean scores were used to divide the participants into two groups; good, and poor. For attitudinal and practice questions respondents who scored above the mean was considered as having good attitude and practice and less than the mean score as poor attitude and practice.

The completed questionnaires were checked for completeness, edited sorted and entered into EpiData version 3.1 and exported to version 20 of Statistical Package for the Social Sciences (SPSS) for analysis. The data was explored using descriptive and frequencies to clean data. Scatter plots, skewness, and kurtosis were examined to determine the normality of the data distribution. On the basis of this information, data distribution was determined, and for those not normally distributed median was taken. The assumption of logistic regressions was checked. Then, Binary logistic regression analysis was done to see the independent effect of predictors on the dependent variables and predictors with P-valve ≤ 0.25 were entered in the multivariable logistic regression analysis model to identify final predictors of knowledge, attitude and practice level after controlling other independent variables. Odds ratio and 95% CI were calculated and P≤0.05 was considered statistically significant. Finally, the result was described in text form and summarized and presented in tables and graphs.

To keep the quality of data pre-testing of the questionnaire was done, proper training of the data collectors on the data collection procedure, checking the completeness of the data at field level and repeated revisits were done to get participants in case of absence. To avoid data entry error, double data entry through EpiData version 3.1 was used proper categorization and coding of data was done during data cleaning phases.

## Result

Five hundred and ninety four (594) respondents were participated and gave a response rate of 98.2%.

### Socio demographic characteristics of respondents

Of the 594 respondents, 327 were females (55.1%) aged 18–80 years (median 28 years) while 267 were males, 44.9% were aged between 18–85 years (median 30 years). More than half of the respondents (61%) were married, and had education levels between grade nine and twelve (37.2%). Almost one thirds were housewives (27%). Aside this, one third of them had an average family income that ranged between 1001–2000 Ethiopian birr. Around 90% of the participants heard about diabetes mellitus and their sources of information were the media (37%). Of this 84% sources of information were television or radio. Eleven percent of participants had family history of diabetes.**(Table 1).**

**Table 1:** Socio demographic characteristics of respondent’s to assess knowledge, attitude and practice and associated factors towards diabetes mellitus among community members of Bale zone administrative towns, South East Ethiopia, 2015 (N = 594).

Participant’s characteristics			No	%
**Address category**	Goba		178	30
	Ginnir		106	17.8
	Robe		310	52.2
**Gender**	Male		267	44.9
	Female		327	55.1
**Age category**	≤24		179	30.1
	25–34		191	32.2
	35–44		123	20.7
	>44		101	17
**Marital status**	Single		188	31.6
	Married		363	61.1
	Divorced /separated		21	3.5
	Widowed		22	3.7
**Level of education**	unable to read &write		43	7.2
	able to read and write		27	4.5
	Grade 1–4		56	9.4
	Grade 5–8		127	21.4
	Grade 9–12		221	37.2
	College & above		120	20.2
**Occupation**	House wife		164	27.6
	Student		87	14.6
	Merchant		136	22.9
	Farmer		47	7.9
	Government/private employee		91	15.3
	Daily laborer		47	7.9
	Other*		20	3.4
**Average family income category (EBR)**	≤500		148	24.9
	501–1000		137	23.1
	1001–2000		156	26.3
	>2000		153	25.8
**Have you heard about DM**	Yes		531	89.4
	No		63	10.6
**Sources of information about DM**	Media	Yes	220	37
		No	374	63
	Health care workers	Yes	70	11.8
		No	524	88.2
	Friends/relatives	Yes	213	35.9
		No	381	64.1
	Others**	Yes	52	8.8
		No	542	91.2
**Family history of DM**	Yes		66	11.1
	Do not Know		46	7.7
	No		482	81.1
**Exposure to DM health education**	Yes		91	15.3
	No		503	84.7
**Have television/radio**	Yes		497	83.7
	No		97	16.3

*Brokers, Drivers.

**Teachers, Religious leaders.

### Knowledge of participants towards diabetes mellitus

From **(Table 2),** participants responded correctly as diabetes affected part of body (49.5%), and defined as high levels of sugar in the blood(49%) and is incurable (40.2%). Participants stated that being overweight and /or Obesity (60.3%) and not getting enough exercise (55.6%) could predispose them to develop diabetes. Regarding signs and symptoms of diabetes, excessive hunger (79.6%) and feeling of weakness (73.4%) are highly rated. They also described as diabetes can be controlled by insulin injection (70%) and medical eye checkup and care (64.6%). Eye problem or even blindness (43.9%) and heart failure (39.2%) were major complication of diabetes identified by the participants.

**Table 2:** Frequency distribution of participants response of knowledge towards diabetes mellitus, Bale Zone administrative towns, 2015(n = 594).

Variables	Yes		NO		I don’t know	
	No	%	No	%	No	%
**What is/are DM**						
DM is a condition of insufficient insulin production	163	27.4	43	7.2	388	65.3
DM is a condition of the body which not responding for insulin	159	26.8	47	7.9	388	65.3
DM is a condition of high level of sugar in the blood	291	49	37	6.2	266	44.8
DM is not curable	239	40.2	182	30.6	173	29.1
DM is diseases which affect any part of body	294	49.5	122	20.5	178	30
**What are the risk factors of DM**						
Older age	180	30.3	203	34.2	211	35.5
Genetic or family history of diabetes mellitus	183	30.8	191	32.2	220	37
Being overweight and /Obesity	358	60.3	82	13.8	154	25.9
Pregnancy	178	30	118	19.9	298	50.2
Sedentary life /Poor dietary habits	238	40.1	147	24.7	209	35.2
Not getting enough exercise can predispose to diabetes	330	55.6	97	16.3	167	28.1
**What are signs and symptoms of DM**						
Frequent urination	279	47	68	11.4	247	41.6
Excessive thirst	336	56.6	61	10.3	197	33.2
Excessive hunger	473	79.6	37	6.2	84	14.1
Weight loss	244	41.1	152	25.6	198	33.3
High blood sugar	317	53.4	65	10.9	212	35.7
Blurred vision	245	41.2	85	14.3	264	44.4
Slow healing of cuts and wounds	249	41.9	79	13.3	266	44.8
Feeling of weakness	436	73.4	41	6.9	117	19.7
**Control and management of DM**						
Insulin injection is available for control and management of Dm	416	70	33	5.6	145	24.4
Tablets & capsule are available for control and management of DM	309	52	58	9.8	227	38.2
Regular Exercise	331	55.7	80	13.5	183	30.8
Practices healthy diet	348	58.6	73	12.3	173	29.1
Medical eye checkup and care	384	64.6	74	12.5	136	22.9
Feet and toes medical checkup and care	371	62.5	67	11.3	156	26.3
Weight reduction	354	59.6	70	11.8	170	28.6
**Complications of DM(62.76%)**						
Diabetes can cause eye problem or even blindness	261	43.9	77	13	256	43.1
Diabetes can cause kidney failure	224	37.7	71	12	299	50.3
Diabetes can cause heart failure	233	39.2	65	10.9	296	49.8
Diabetes can cause brain disease like Stroke	173	29.1	75	12.6	346	58.2
Diabetes can result in Amputation of limb	214	36	94	15.8	286	48.1

The total mean score for correctly answered knowledge questions were (14.86 ±7.9). Three hundred twelve participants (52.5%) scored mean and above the mean were considered to be knowledgeable while 282 (47.5%) scored below the mean and considered as not knowledgeable. Participants had less frequency on signs and symptoms knowledge questions of diabetes (47.6%) and had frequency above half in diabetes definition, control and management, and its complication related questions**(Table 3).**

**Table 3:** Mean and percentage distribution of participant’s diabetic knowledge response for deferent components of diabetes knowledge questions, Bale Zone administrative towns, 2015(n = 594).

Response	Mean	Knowledge level				
		Knowledgeable			Not Knowledgeable	
		No	%	95% CI	No	%
What is/are DM	1.93	324	54.5	50.5,58.6	270	45.5
What are the risk factors of DM	2.47	285	48	43.9,52	309	52
What are signs and symptoms of DM	4.34	283	47.6	43.6,51.6	311	52.4
Control and management DM	4.23	312	52.5	48.5,56.6	282	47.5
Complications of DM	1.86	306	51.5	47.5,55.6	288	48.5
**Over all diabetic knowledge level**	**14.83**	**312**	**52.5**	**48.5, 56.5**	**282**	**47.5**

### Attitude of participants towards diabetes mellitus

Almost 44% rated with strong agreement on “do you think that you should be examined for DM and their family members should be screened for DM, while 66% strongly disagreed with the question being asked as “do you discuss stopping smoking with your healthcare team” **(Table 4).**

**Table 4:** Frequency distributions of respondents of attitude towards diabetes mellitus, Bale Zone administrative towns, 2015 (n = 594).

Question	Response option									
	Strongly disagree		Disagree		Neutral		Agree		Strongly agree	
	No	%	No	%	No	%	No	%	No	%
I don’t mind if others know that I am with DM	28	4.7	106	17.8	17	2.9	224	37.7	219	36.9
Do you think that you should be examined for DM	15	2.5	36	6.1	21	3.5	260	43.8	262	44.1
Do you think family members should be screened for DM	8	1.3	31	5.2	24	4.0	270	45.5	261	43.9
Do you think support from family and friends is important in dealing wit	19	3.2	37	6.2	41	6.9	276	46.5	221	37.2
Do you think should we follow avoiding of consumption of too much sugar	29	4.9	63	10.6	60	10.1	256	43.1	186	31.3
DM is not seriously affects the marital relationship	44	7.4	171	28.8	112	18.9	175	29.5	92	15.5
I don’t think DM seriously affect daily activities	29	4.9	187	31.5	67	11.3	213	35.9	98	16.5
Do you think physical activity can prevent risk of DM	24	4.0	86	14.5	77	13.0	284	47.8	123	20.7
Do you discuss stopping smoking with your healthcare team	66	11.1	109	18.4	232	39.1	116	19.5	71	12.0
Do you think maintaining a healthy weight is important in management of	33	5.6	86	14.5	91	15.3	266	44.8	118	19.9
DM complications may be prevented if blood glucose level is well control	35	5.9	61	10.3	103	17.3	266	44.8	129	21.7

The mean score of the participant’s attitude was 1.56 ±0.5. Participants who scored below the mean score were 262 (44.1% which was considered poor attitude) and above the mean score 332 (55.9% which was considered good attitude).

### Practice level of participants towards diabetes mellitus

Around 41% of the participants did not check their blood pressure at all, 38.2% consumed fatty foods frequently, and 31.8% did 30–60 minutes physical activities very frequently**(Table 5).**

**Table 5:** Frequency distributions of respondents of practice towards diabetes mellitus, Bale Zone administrative towns, 2015 (n = 594).

Practice Question	Response option									
	Not at all		Less frequent		Not sure		Frequent		Very frequent	
	No	%	No	%	No	%	No	%	No	%
Do you consume of fatty foods?	52	8.8	224	37.7	37	6.2	227	38.2	54	9.1
Do you do 30–60 minutes physical activity daily? E.g. Brisk walking, house	56	9.4	127	21.4	48	8.1	174	29.3	189	31.8
Do you participate in maintaining your healthy weight?	151	25.4	188	31.6	45	7.6	147	24.7	63	10.6
Do you drink alcohol and smoke tobacco?	401	67.5	71	12.0	58	9.8	44	7.4	20	3.4
Do you check your blood sugar?	241	40.6	220	37.0	26	4.4	65	10.9	42	7.1

The mean score of the participant’s practice level was 1.57 ±0.5. Those participants who scored below the mean score were 258 (43.4% which was considered as poor practice) and above the mean score 336 (56.6% which was considered as good practice).

### Factors associated with participant’s knowledge level towards DM

Among variable entered in the bi-variate analysis participants, gender, level of education, average monthly income category, hearing about diabetes, family history of diabetes, and exposure to diabetes health education and having televisions/radios showed significant associations. Variables with P-value ≤ 0.25were entered in the multivariable logistic analysis and some of the above association did not exist after adjustment for other variables. In the multivariable logistic analysis, subjects earning average monthly family income of ≤500 Ethiopian Birr were 0.4 times (AOR = 0.4, CI = 0.2,0.6) and 501–1000 (AOR = 0.4, CI = 0.2,0.7) Ethiopian Birr were 0.4 times less likely to have diabetes knowledge as compared to those earned ≥ 2000 Ethiopian Birr. Individuals who have heard about diabetes had 4.4 times (AOR = 4.4, CI = 1.9, 10.2) more likely to have diabetes knowledge as compared to those who did not hear. Regarding diabetes health education exposure history, subjects who had exposure had 5 times (AOR = 5,CI = 2.5, 9.7) more likely to have diabetes knowledge as compared to those who did not have diabetes health education exposure (**Table 6).**

**Table 6:** Bivariable and multivariable logistic regression predicting diabetes mellitus related knowledge among community members of Bale Zone Administrative towns, 2015(N = 594).

Variable category	Knowledge level			COR (95% CI)	P-value	AOR (95%CI)	P-value
	Not knowledgeable	knowledgeable					
	No (%)	No	%				
**Gender**							
Male	111(41.6)	156	58.4	1.54(1.11,2.13)	0.009**	1.5(0.98,2.4)	0.059
Female(Ref)	171(52.3)	156	47.7				
**Marital status**							
Single (Ref)	88(46.8)	100	53.2		0.29		
Married	169(46.6)	194	53.4	2.44(0.95,6.25)	0.64		
Divorced/separated	10(47.6)	11	52.4	2.46(0.98,6.18)	0.055		
Widowed	15(68.2)	7	31.8	2.34(0.68,8.15)	0.18		
**Level of education**							
Unable to read & write	30(69.8)	13	30.2	0.19(0.087,0.4)	0.000**	0.4(0.13,1.1)	0.07
Able to read &write	17(63)	10	37	0.25(0.11,0.6)	0.002*	0.5(0.16,1.4)	0.2
Grade 1–4	31(55.4)	25	44.6	0.35(0.18,0.67)	0.001*	1.04(0.4,2.6)	0.94
Grade 5–8	72(56.7)	55	43.3	0.33(0.19,0.55)	0.000**	0.63(0.3,1.3)	0.213
Grade 9–12	96(43.4)	125	56.6	0.56(0.35,0.9)	0.016*	0.94(0.5,1.8)	0.9
College & above(Ref)	36(30)	84	70				
**Average family income**							
≤500	85(57.4)	63	42.6	0.34(0.21,0.54)	0.000**	0.4(0.2,0.6)	0.000**
501–1000	80(58.4)	57	41.6	0.33(0.2,0.53)	0.000**	0.4(0.2,0.7)	0.001*
1001–2000	69(44.2)	87	55.8	0.57(0.36,0.92)	0.02*	0.6(0.4,1.1)	0.1
>2000(Ref)	48(31.4)	105	68.6				
**Have you heard about DM**							
Yes	225(42.4)	306	57.6	6.5(3.14,13.4)	0.000**	4.4(1.9,10.2)	0.001*
No (Ref)	57(90.5)	6	9.5				
**Family history of DM**							
Yes	20(30.3)	46	69.7	2(1.15,3.48)	0.15	1.3(0.7,2.4)	0.5
Do not Know	38(82.6)	8	17.4	0.18(0.08,0.4)	0.000**	0.5(0.16,1.3)	0.14
No (Ref)	224(46.5)	258	53.5				
**Exposure to DM health education**							
Yes	16(17.6)	75	82.4	5.26(2.98,9.28)	0.000**	5(2.5,9.7)	0.000**
No (Ref)	266(52.9)	237	47.1				
**Have television/radio**							
Yes (Ref)	210(42.3)	287	57.7				
No	72(74.2)	25	25.8	3.94(2.42,6.42)	0.000**	0.6(0.3,1.1)	0.095

* For Significant variables

** For highly significant variables

Statistically significant at p<0.05.

### Factors associated with participant’s attitude level towards DM

Educational level category, occupational status, and average monthly family income category, family history of diabetes, exposure to diabetes health education and knowledge level showed significant associations in the bivariate analysis. Some of these associations did not exist after adjustment for other variables.In the multivariable logistic regression analysis, being a student had 5.1 times (AOR 5.1,CI 2.1,12), being merchant had 2 times (AOR = 2,CI = 1.1,3.6), being government/private employee had 3 times(AOR = 3,CI = 1.4,6.7) more likely to have positive attitude towards DM as compared to being house wives. Individuals who had earned ≤ 500 Ethiopian Birr had 0.5 times (AOR = 0.5, CI = 0.3, 0.85) less likely to have positive attitude towards DM as compared to those who earned ≥2000 Ethiopian Birr. Regarding diabetes knowledge level, knowledgeable subjects had 3 times (AOR = 3, CI = 2.1, 4.7) more likely to have positive attitude towards diabetes as compared to those who were not knowledgeable **(Table 7).**

**Table 7:** Bi- variable and multivariable logistic regression predicting diabetes mellitus related attitude level among community members of Bale Zone Administrative towns, 2015(N = 594).

Variable category	Attitude level		COR(95% CI)	P-value	AOR(95%CI)	P-value
	Poor	Good				
	NO (%)	NO (%)				
**Marital status**						
Single (Ref)	72(38.3)	116(61.7)				
Married	170(46.8)	193(53.2)	0.71(0.5,1)	0.06		
Divorced/separated	11(52.4)	10(47.6)	0.56(0.23,1.4)	0.22		
Widowed	9(40.9)	13(59.1)	0.9(0.37,2.2)	0.81		
**Level of education**						
Unable to read & write	32(74.4)	11(25.6)	0.15(0.07,0.32)	0.000**	0.4(0.1,0.9)	0.048*
Able to read &write	11(40.7)	16(59.3)	0.62(0.26,1.48)	0.28	1.6(0.55,4.4)	0.4
Grade 1–4	22(39.3)	34(60.7)	0.66(0.34,1.29)	0.22	1.9(0.8,4.7)	0.2
Grade 5–8	65(51.2)	62(48.8)	0.41(0.24,0.69)	0.001*	1.1(0.54,2.2)	0.8
Grade 9–12	96(43.4)	125(56.6)	0.56 (0.35,0.9)	0.02	1(0.6,2)	0.84
College & above(Ref)	36(30)	84(70)	1			
**Occupation**						
House wife(Ref)	96(58.5)	68(41.5)				
Student	26(29.9)	61(70.1)	3.31 (1.9,5.77)	0.00**	5.1(2.2,12)	0.00**
Merchant	59(43.4)	77(56.6)	1.84(1.16,2.92)	0.009*	2(1.1,3.6)	0.021*
Farmer	26(55.3)	21(44.7)	1.14(0.59,2.19)	0.69	1.2(0.5,2.8)	0.63
Government/private employee	24(26.4)	67(73.6)	3.94(2.25,6.9)	0.00**	3(1.4,6.7)	0.005*
Daily laborer	21(44.7)	26(55.3)	1.75(0.91,3.36)	0.094	2.3(1.03,5)	0.043*
Other(specify)	9(45)	11(55)	1.73(0.68,4.39)	0.25	1.7(0.6,5)	0.312
**Average family income**						
≤500	86(58.1)	62(41.9)	0.38(0.24,0.61)	0.00**	0.5(0.3,0.85)	0.011*
501–1000	65(47.4)	72(52.6)	0.59(0.37,0.94)	0.027*	0.8(0.5,1.5)	0.6
1001–2000	58(37.2)	98(62.8)	0.9(0.56,1.43)	0.64	1.2(0.7,2)	0.55
>2000(Ref)	53(34.6)	100(65.4)				
**Family history of DM**						
Yes	19(28.8)	47(71.2)	2.06(1.17,3.16)	0.012*	1.6(0.82,3)	0.17
Do not Know	24(52.2)	22(47.8)	0.76(0.42,1.4)	0.38	0.62(0.27,1.4)	0.25
No (Ref)	219(45.4)	263(54.6)	1			
**Exposure to DM health education**						
Yes	26(28.6)	65(71.4)	2.2(1.36,3.6)	0.001*	1.05(0.6,1.9)	0.88
No (Ref)	236(46.9)	267(53.1)				
**Knowledge level**						
Not knowledgeable (Ref)	166(58.9)	116(41.1)	1			
Knowledgeable	96(30.8)	216(69.2)	3.2(2.3,4.5)	0.00**	3(2.1,4.7)	0.00**

* For Significant variables

** For highly significant variables

Statistically significant at p<0.05.

### Factors associated with participants practice level towards DM

Gender, level of education, average monthly family income category, hearing about diabetes, and diabetes attitude levels showed significant associations in the bivariate logistic regression analysis.In the multivariable analysis, individuals with grade 5–8 educational level were 0.33 times (AOR = 0.33, CI = 0.16, 0.7) less likely to practice than those with college and above educational levels. In terms of occupation, farmers practiced 2.5 times (AOR = 2.5, CI = 1.13, 6.14) more likely to practice than housewives. Having good attitude towards diabetes had two times (AOR = 2, CI = 1.3, 3) more likely to practice than those having poor attitude**(Table 8).**

**Table 8:** Bivariable and multivariable logistic regression predicting diabetes mellitus related practice level among community members of Bale Zone Administrative towns, 2015(N = 594).

Variable category	Practice level			COR(95% CI)	P-value	AOR(95%CI)	P-value
	Poor	Good					
	No (%)	No (%)					
**Gender**							
Male	97(36.3)	170	63.7	1.7(1.22,2.37)	0.002*	1.3(0.83,2)	0.3
Female(Ref)	161(49.2)	166	50.8				
**Age category**							
≤24(Ref)	83(46.4)	96	53.6				
25–34	76(39.8)	115	60.2	1.31(0.87,1.98)	0.202		
35–44	45(36.6)	78	63.4	1.5(0.94,2.4)	0.092		
>44	54(53.5)	47	46.5	0.75(0.46,1.23)	0.25		
**Marital status**							
Single (Ref)	81(43.1)	107	56.9				
Married	153(42.1)	210	57.9	1.04(0.73,1.48)	0.83		
Divorced/separated	11(52.4)	10	47.6	0.69(0.28,1.7)	0.42		
Widowed	13(59.1)	9	40.9	0.52(0.21,1.29)	0.16		
**Level of education**							
Unable to read & write	27(62.8)	16	37.2	0.3(0.14,0.61)	0.001*	0.5(0.2,1.3)	0.16
Able to read and write	11(40.7)	16	59.3	0.73(0.31,1.71)	0.47	0.62(0.2,1.85)	0.4
Grade 1–4	21(37.5)	35	62.5	0.83(0.43,1.61)	0.59	0.73(0.3,1.9)	0.52
Grade 5–8	67(52.8)	60	47.2	0.45(0.27,0.75)	0.002*	0.33(0.16,0.7)	0.003*
Grade 9–12	92(41.6)	129	58.4	0.70(0.44,1.12)	0.13	0.8(0.4,1.5)	0.43
College & above(Ref)	40(33.3)	80	66.7				
**Occupation**							
House wife(Ref)	80(48.8)	84	51.2				
Student	39(44.8)	48	55.2	0.17(0.7,2)	0.55		
Merchant	57(41.9)	79	58.1	1.32(0.84,2.09)	0.24		
Farmer	16(34)	31	66	1.85(0.94,3.63)	0.08	2.7(1.13,6.14)	0.025*
Government/private employee	38(41.8)	53	58.2	1.33(0.79,2.23)	0.28		
Daily laborer	25(53.2)	22	46.8	0.84(0.44,1.61)	0.6		
Other(specify)	3(15)	17	85	5.4(1.52,19.12)	0.009*		
**Average family income**							
≤500	62(41.9)	86	58.1	0.9(0.57,1.42)	0.64		
501–1000	71(51.8)	66	48.2	0.6(0.78,0.96)	0.032*		
1001–2000	65(41.7)	91	58.3	0.9(0.57,1.42)	0.66		
>2000(Ref)	60(39.2)	93	60.8				
**Have you heard about DM**							
Yes (Ref)	216(40.7)	315	59.3				
No	42(66.7)	21	33.3	0.34(0.2,0.6)	0.00**	0.43(0.2,0.92)	0.013*
**Family history of DM**							
Yes	29(43.9)	37	56.1	0.89(0.53,1.49)	0.66		
Do not Know	31(67.4)	15	32.6	0.34(0.18,0.64)	0.001*	0.35(0.2,0.8)	0.012*
No (Ref)	198(41.1)	284	58.9				
**Exposure to DM health education**							
Yes	38(41.8)	53	58.2	1.08(0.69,1.7)	0.73		
No (Ref)	220(43.7)	283	56.3				
**Have television/radio**							
Yes (Ref)	207(41.6)	290	58.4				
No	51(52.6)	46	47.4	0.64(0.42,1)	0.048		
**Attitude level**							
Poor attitude (Ref)	132(50.4)	130	49.6				
Good attitude	126(38)	206	62	1.7(1.2,2.3)	0.002*	2(1.3,3)	0.002

* For Significant variables

** For highly significant variables

Statistically significant at p<0.05.

## Discussion

The current study showed, more than 50% of the study participants were knowledgeable (52.5%), had good attitude (55.9%) and practice (56.6%) towards diabetes mellitus. The scores seemed higher than previous studies, having community members with this gap necessitate being seen carefully because of their crucial role in its prevention.

In this study, 52.5%participants were knowledgeable towards diabetes mellitus. This showed higher as compared to community-based studies done in Sudan (15%) [25],Malaysia(41.9%) [17],Mangalore (35%) [18], Tarlai (43%) [19],Kenya (27%)[26], and almost similar score with study done in Debre Tabor town, Ethiopia (49%)[27] and India (49.9%)[38].These differences were probably explained by the studies conducted in Sudan, Malaysia, Tarlai and Mangalore was only in rural communities while both urban and rural communities included in Kenya. The score was lower than the study conducted in Waghodia(56.14%) [22].This is because of the limited organized diabetics education facilitates and less participations of media and NGO in awareness creation about diabetes mellitus as compared to Waghodia.

Almost 52% of participants were not knowledgeable regarding risk factors, sign, and symptoms of diabetes; and more than half were knowledgeable in DM definition (54.5%), control and management (52.5%), and its complication (51.5%).This study was consistent with the study done in Debre Tabor Town communities in Ethiopia which showed more than half (60.3%)knew the definition,61% had poor knowledge about symptoms, 53% were not able to identify the cause /risk factors, 44% had good knowledge about control and management and 57.8% had good knowledge about its complications[27].Also was consistent with a study conducted in Semi-Urban community of Omani population which showed that respondents had good knowledge about definition (46.5%), symptoms (57%) and its complications (55.1%)[12].Study in urban and semi urban population of Peshawar, Pakistan showed almost similar knowledge in symptoms (47.1%), but lower score in identification of complications (30.8%)[23].

In this study respondents had responded correctly to diabetes affect any part of body (49.5%), and defined as high level of sugar in the blood (49%) and is not curable (40.2%).This study finding was higher than a study done in Debre Tabor where participants stated that diabetes mellitus is incurable (51.3%), diabetes affects all parts of the body (43.3%), and diabetes is a condition of high level of sugar in the blood (41.2%)[27].

In this study participants stated that being overweight /Obesity (60.3%) and not getting enough exercise (55.6%), sedentary way of life (40.1%),family history of DM (30.8%) and pregnancy (30%) can predispose an individual to diabetes. Regarding symptoms of diabetes excessive hunger (79.6%) feeling of weakness (73.4%), excessive thirst (56.6%), and high blood sugar (53.4%) were highly rated. This finding was higher as compared to a study done in Debre Tabor in which obesity (35.9%), sedentary life(33.7%), family history of DM(32.6%), older age(26%) and pregnancy (21.9%) were risk factors of DM and frequent hunger (78%), frequent thirst (48%) are symptoms of DM. In addition, insulin injection (70%) and medical eye checkup and care (64.6%), proper diet (58%), feet and toes medical checkup and care (62.5%), weight reduction (59.6%) and regular exercise (55.7%) can help to control DM. Blindness (43.9%) and heart failure (39.2%),kidney failure (37.7%),brain disease (29.1%) and limb amputation (36%) were major complications of DM. These finding were supported by the study done in Debre Tabor, which showed that insulin injection(57.3%), practicing healthy diet (56%) were ways of controlling and managing diabetes mellitus. Moreover, limited knowledge were seen on complications of DM like brain diseases (47.5%), blindness (35.3%), amputation of limb (33.2%) and kidney problem (29.3%)[27].

The differences might be due to inadequate levels of information, limited sources of information, inadequate involvements of media and other concerned body to community on risk factors and consequences of diabetes in Ethiopia as compared to other countries.

The current study also showed 55.9% had good attitude towards diabetes. The findings is higher as compared to a study conducted in Debre Tabor (41.4%), Kenya (49%) Waghodia, India (17.6%) [22,27,26]. These differences could be explained by study participants in Kenya and Waghodia were from rural areas only, while in Debre Tabor participants were only from one town, but in this study participants were from three towns. Around 57% of participants had good practice towards diabetes mellitus. This finding showed higher as compared to study done in Kenya (41%) [26]. This could be due to study in Kenya which included both urban and rural communities which might have different information level.

Having higher family monthly income, ever hearing about diabetes and exposure to diabetes health education were the determining factors of diabetes Knowledge in this study.

Respondents whose family income per month were ≥ 2000 Ethiopian Birr had 0.4 fold increase in diabetes knowledge level as compared to those having family income per month ≤ 500 Ethiopian Birr. These findings were supported by studies conducted in Debre Tabor, Malaysia and India which stated that those who belonged to the upper socioeconomic strata had more knowledge towards DM [17,20,27]. This could be explained by participants who had low income, cannot afford checkups in private clinics without waiting the regular consultation time arranged by health institution and by community.Resources are necessarily for sustained life style modification or behavioral change and lack of resources could be a barrier for life style modification.

Participants who had ever heard about diabetes had 4.4 fold increases in their diabetes knowledge level as compared to subjects did not hear before. The finding was supported by the finding conducted in Bangladesh in which respondents who get information regarding diabetes scored significantly higher than the group who did not get any information[16].

Subjects who had diabetes health education exposure history were a5 fold knowledgeable as compared to subjects who did not have diabetes health education exposure. This finding was consistent with the study conducted in Bangladesh in which respondents who did not received any information regarding diabetes scored lower than the respondents who get information[16].

Good attitude was observed 5.1 fold increases in students, 3 fold increases in government/private employee and 2 fold increases in merchants as compared to house wives. The finding was consistent with the study conducted in Bangladesh in which the attitude score was significantly lower in housewives respondents than the respondents of other occupations [16].Good attitude was observed in subjects whose families had higher monthly incomes. These might be because of having higher income level will help to access and afford necessary information related to diabetes that resulted in changed behavior among the participants.

Being knowledgeable about diabetes had 3 fold increases in their positive attitude level about diabetes as compared to those who were not knowledgeable. These finding supported the idea about positive correlation between knowledge and good attitude was observed among participants in a study conducted in Malaysia [32].

Respondents having college and higher educational levels had seven fold increase of attitude as compared to grade 5-8.Probably, because respondents who had higher education would have the chance to get different information contented materials like leaflet and manuals which make them more aware about diabetes. Even they can communicate health care providers easily if they have any doubts.

The current study found that farmers had 2.5 fold increases in their practice level than housewife. It is expected that farmers spend much time on manual works than housewife and they do more physician activities even though the activities are not planned, and in developing countries like Ethiopia, females are lagging behind in all aspects.

The present study found that there was significant positive associations between attitude and practice level i.e. having good attitude towards diabetes had a 2-fold increase in their practice level. The study indicated that individuals with positive attitude towards diabetes will implement diabetes risk reduction activities easily.

## Strength and limitation of the study

The strength of the study was (1) large sample size, (2) community-based recruitment strategies and approach,(3) areas assessed were interested because little is known by the population, (4) tried to identify those with clinical confirmed diabetes because such people would have higher knowledge due to the clients education provided at the diabetes clinic. The limitation of the study was exclusion of homeless individuals and some of the result was compared with studies conducted on diabetic patient due to limited findings at community level.

## Implication for practice

As shown above and by different literatures mentioned, knowing levels and factors associated with knowledge, attitude, and practice towards diabetes mellitus are important to deliver appropriate diabetes information to the community. As we keep the community aware on diabetes, the community can participate in the prevention and management of diabetes while they develop the disease.

## Conclusion and recommendation

In general, there were a considerable limited knowledge attitude and practice about diabetes in the population of Bale Zone administrative town populations particularly diabetes symptoms and risk factor modifications.

The zonal health bureau, Woreda’s health offices could integrate non communicable disease like DM with the health extension packages might helps to create awareness in the community through health education. This will help in controlling diabetes through promote on early screening, diagnosis, and initiating of effective treatment which will result in preventing diabetes associated complication and disabilities. Since medias was the source of information in majority of the participants, great emphasis on diabetes signs and symptoms, and risk factor modification has to be given to it. Awareness meetings have to be conducted by incorporating various targeted population groups such as health bureau officers, health extension workers schoolchildren, youth, nongovernmental organizations/agencies. Leaflets, posters and banners about awareness have to be used. Madda Walabu University has to prepare a zonal diabetic awareness campaign which will be held on same day of International Diabetes Day in each year. To make the campaign interesting, it has to be done not only at health facilities, but also in work places and to the public through public campaigns and seminars.

## Supporting information

S1 File Supplemental file questionnaires. (DOCX)
