# Supplementary material for: Knowledge, attitude, practices and their associated factors towards diabetes mellitus among non diabetes community members of Bale Zone administrative towns, South East Ethiopia. A cross-sectional study
Source: PLoS One. 2017 Feb 2;12(2):e0170040. doi: 10.1371/journal.pone.0170040 (PMC5289457; doi:10.1371/journal.pone.0170040)
Supplement: S1 File — (DOCX) [file pone.0170040.s001.docx]

**S1 Table: English version of the questionnaires**

**Questionnaire ID No.__________**

**Madda Walabu University**

**College of medicine and health Sciences**

**Department of Nursing**

A **questionnaire** prepared to collect data to assess Knowledge, attitude and practice and its associated factors towards diabetes mellitus among community members of bale zone administrative towns, South East Ethiopia, 2015.

Dear Mr. /Ms. -----------------------------

I am ---------------------------- and currently working as lecturer at Madda Walabu University College of medicine and health sciences, Department of Nursing. This research topic is aimed to assess the Knowledge, attitude and practice and its associated factors related to diabetes mellitus among community members of bale zone administrative towns, South East Ethiopia, 2015.The community may be at different levels in terms of diabetes knowledge, attitude and practice and its associated factors. This is a situation that needs to be addressed. The results of this study will produce information that will be useful in preventing diabetes. The study will involve your information and it will not take more than 20 minutes to finish. Confidentiality and anonymity is fully assured, as your name is not required and only the research team will have access to the results. It will not affect you in anyway, should you not take part in this study.

**Consent Form**

I have been informed that the purpose of this study is to assess knowledge attitude and practice and its associated factors related to diabetes among community members of Bale zone administrative towns, Ethiopia, 2015.

I have understood that participation in this study is entirely voluntarily. I have been told that my answers to the questions will not be given to anyone else and no reports of this study ever identify me in any way. I have also been informed that my participation or non-participation or my refusal to answer questions will have no effect on me. I understood that participation in this study does not involve risks.

I understood that Chanyalew Worku Kassahun is the contact person if I have questions about the study or about my rights as a study participant.

**Address of the principal investigator:**

Name: Chanyalew Worku Kassahun

Cell phone: +251 921252375

E-mail: chanyalewworku@gmail.com

**Respondent’s Signature___________Date___________.**

**Start your interview. Date: ________Time started: _______Time finished: _________.**

**Supervisor’s name ________________ signature _________**

**Instruction: Please circle the number in front of the option the respondents choose & fill in the blank space that the respondent best describe in the right side of the table**.

| **Part-I: Respondent’s socio- demographic information** | | | |
| --- | --- | --- | --- |
| **S.No** | **Questions** | **Response options** | **Remarks** |
| 100 | Sex | **0** = Male  **1** = Female |  |
| 101 | Age | ________Years old |  |
| 102 | Marital status | **0** = Single  **1** = Married  **2** = Divorced/Separated  **3** = Widowed |  |
| 103 | Level of education | **0** = Unable to read and write  **1** = Able to read and write  **2** = Grade 1-4  **3** = Grade 5-8  **4** = Grade 9-12  **5** = College and above |  |
| 104 | Occupation | 0= House wife  1 = Husband  2= Student  3 = Merchant  4 = Farmer  5 = Government/private employee  6= Daily laborer  7= Other(specify)_____________ |  |
| 105 | Average family monthly income | _______________(in Ethiopian birr) |  |
| 106 | Have heard the disease “DM”? | 0 = Yes  1 = No |  |
| 107 | Exposure to health education about DM | 0 = Yes  1 = No |  |
| 108 | Have television/radio | 0 = Yes  1 = No |  |
| 109 | Family history of DM | 0 = Yes  1 = No  2 = Don’t know |  |
| 110 | If “Yes” for Q-106 your sources of information? | **0** = Medias  **1** = Health care workers  **2** = Friends/relatives  **3** = Others (teacher, religious leader…) |  |

| **Part-II: Knowledge questions related to DM** | | | | | |
| --- | --- | --- | --- | --- | --- |
| **S.No** | **Questions** | **Response options** | | | |
|  |  | **Yes =1** | **No**  **= 2** | **Do not know**  **= 3** | **Remarks** |
|  | **What is/are DM** | **1** | **2** | **3** |  |
| 200 | DM is a condition of insufficient insulin production | **1** | **2** | **3** |  |
| 201 | DM is a condition of the body which not responding for insulin | **1** | **2** | **3** |  |
| 202 | DM is a condition of high level of sugar in the blood | **1** | **2** | **3** |  |
| 203 | DM is not curable | **1** | **2** | **3** |  |
| 204 | DM is diseases which affect any part of body | **1** | **2** | **3** |  |
|  | **What are the risk factors of DM** |  |  |  |  |
| 205 | Older age | **1** | **2** | **3** |  |
| 206 | Genetic or family history of diabetes mellitus | **1** | **2** | **3** |  |
| 207 | Being overweight /Obesity | **1** | **2** | **3** |  |
| 208 | Pregnancy | **1** | **2** | **3** |  |
| 209 | Sedentary life /Poor dietary habits | **1** | **2** | **3** |  |
| 210 | Not getting enough exercise can predispose to diabetes | **1** | **2** | **3** |  |
|  | **What are signs and symptoms of DM** |  |  |  |  |
| 211 | Frequent urination | **1** | **2** | **3** |  |
| 212 | Excessive thirst | **1** | **2** | **3** |  |
| 213 | Excessive hunger | **1** | **2** | **3** |  |
| 214 | Weight loss | **1** | **2** | **3** |  |
| 215 | High blood sugar | **1** | **2** | **3** |  |
| 216 | Blurred vision | **1** | **2** | **3** |  |
| 217 | Slow healing of cuts and wounds | **1** | **2** | **3** |  |
| 218 | Feeling of weakness | **1** | **2** | **3** |  |
|  | **Control and management DM** |  |  |  |  |
| 218 | Insulin injection is available for control and mgt of Dm | **1** | **2** | **3** |  |
| 219 | Tablets & capsule are available for control and management of DM | **1** | **2** | **3** |  |
| 220 | Regular Exercise | **1** | **2** | **3** |  |
| 221 | Practices healthy diet | **1** | **2** | **3** |  |
| 222 | Medical eye check up and care | **1** | **2** | **3** |  |
| 223 | Feet and toes medical checkup and care | **1** | **2** | **3** |  |
| 224 | Weight reduction | **1** | **2** | **3** |  |
|  | **Complications of DM** |  |  |  |  |
| 225 | Diabetes can cause eye problem or even blindness | **1** | **2** | **3** |  |
| 226 | Diabetes can cause kidney failure | **1** | **2** | **3** |  |
| 227 | Diabetes can cause heart failure | **1** | **2** | **3** |  |
| 228 | Diabetes can cause brain disease like Stroke | **1** | **2** | **3** |  |
| 229 | Diabetes can result in Amputation of limb | **1** | **2** | **3** |  |

| **Part-III: Attitude question of the respondents towards diabetes** | | | | | | | | | | | | | |
| --- | --- | --- | --- | --- | --- | --- | --- | --- | --- | --- | --- | --- | --- |
| **S.No** | **Questions** | | | **Response options** | | | | | | | | | |
|  |  |  |  | Strongly Agree | | Agree | | Neutral | | Disagree | | Strongly  disagree | Remarks |
| 300 | I don’t mind if others know that I am with DM | | | **5** | | **4** | | **3** | | **2** | | **1** |  |
| 301 | Do you think that you should be examined for DM | | | **5** | | **4** | | **3** | | **2** | | **1** |  |
| 302 | Do you think family members should be screened for DM | | | **5** | | **4** | | **3** | | **2** | | **1** |  |
| 303 | Do you think support from family and friends is important in dealing with DM | | | **5** | | **4** | | **3** | | **2** | | **1** |  |
| 304 | Do you think should we follow avoiding of consumption of too much sugar for controlling of DM | | | **5** | | **4** | | **3** | | **2** | | **1** |  |
| 305 | DM is not seriously affects the marital relationship | | | **5** | | **4** | | **3** | | **2** | | **1** |  |
| 306 | I don’t think DM seriously affect daily activities | | | **5** | | **4** | | **3** | | **2** | | **1** |  |
| 307 | Do you think physical activity can prevent risk of DM | | | **5** | | **4** | | **3** | | **2** | | **1** |  |
| 308 | Do you discuss stopping smoking with your healthcare team | | | **5** | | **4** | | **3** | | **2** | | **1** |  |
| 309 | Do you think maintaining a healthy weight is important in management of diabetes | | | **5** | | **4** | | **3** | | **2** | | **1** |  |
| 310 | DM complications may be prevented if blood glucose level is well controlled | | | **5** | | **4** | | **3** | | **2** | | **1** |  |
|  |  | | |  | |  | |  | |  | |  |  |
| **Part-IV: Practice questions relating to DM in terms of adopting healthy lifestyles that promote diabetes prevention** | | | | | | | | | | | | | |
| **S.No** | | **Questions** | **Response options** | | | | | | | | | | |
|  |  |  | Very  frequent  = 5 | | Frequent = 4 | | Not sure =3 | | Less frequent = 2 | | Not at all =1 | | Remarks |
| 400 | | Do you consume of fatty foods? |  | |  | |  | |  | |  | |  |
| 401 | | Do you do 30-60 mins physical activity daily? E.g. Brisk walking, house activities, climbing staircase. | **5** | | **4** | | **3** | | **2** | | **1** | |  |
| 402 | | Do you participate in maintaining your healthy weight? | **5** | | **4** | | **3** | | **2** | | **1** | |  |
| 403 | | Do you drink alcohol and smoke tobacco? | **5** | | **4** | | **3** | | **2** | | **1** | |  |
| 404 | | Do you check your blood sugar regularly? | **5** | | **4** | | **3** | | **2** | | **1** | |  |

**Thank you very much!!!!**

**S2 Table: Amharic version of the questionnaires**

**የመጠይቅ መለያ ቁጥር __________**

**መዳወላቡ ዩኒቨርሲቲ**

**ጎባ ሪፈራል ሆስፒታል**

**ጤና ሳይንስ ት/ቤት**

**የነርሲንግ ት/ት ክፍል**

**መጠይቅ**

ይህ መጠይቅ የተዘጋጀዉ በ2008 ዓ.ም በባሌ ዞን በሚገኙ የከተማ መስተዳደሮች በሚገኙ ህብረተሰቦች መካከል ስለ ስኳር በሽታ ያላቸዉን የእዉቀት፣የአመለካከት እና የተግባር ደረጃ ለማወቅ እና እነዚህን የሚወስኑ ነገሮችን ለመለየት ነዉ፡፡

ዉድ አቶ/ወሮ፡- -----------------------------

እኔ ስሜ ---------------------------- ይባላል፡፡በአሁኑ ሰዓት በመዳወላቡ ዩኒቨርሲቲ ጎባ ሪፈራል ሆስፒታል ጤና ሳይንስ ት/ቤት ነርሲንግ ት/ት ክፍል በመምህርነት እያገለገልኩ እገኛለሁ፡፡ይህ የምርምር ዋና አላማዉ በባሌ ዞን በሚገኙ የከተማ መስተዳደሮች በሚገኙ ህብረተሰቦች መካከል ስለ ስኳር በሽታ ያላቸዉን የእዉቀት፣የአመለካከት እና የተግባር ደረጃ ለማወቅ እና እነዚህን የሚወስኑ ነገሮችን ለይቶ ለማጥናት ነዉ፡፡ህብረተሰቦቹ ስለ ስኳር በሽታ ያላቸዉ የእዉቀት፣የአመላካከት እና ተግባር ደረጃ የተለያየ ሊሆን ይችላል፡፡ይህ ሁኔታ ደግሞ በጥናት መረጋገጥ ይፈልጋል፡፡

የዚህ ጥናት ዉጤት የስኳር በሽታን ለመከላከል የሚያግዙ ሃሳቦችን ለማመንጨት ያግዛል፡፡ በዚህ ጥናት ከአንተ የሚጠበቀዉ ያለህን/ሽን ሃሳብ ማካፈል ነዉ፡፡ከ 20 ደቂቃ በላይ አይወስድብህም፡፡ የሰጠኸዉ/ሽዉ ሃሳብ ሚስጢራዊነቱ የተጠበቀ እና ማን ሃሳብ እንደሰጠ የማይታወቅ ነዉ፡፡የተሰጠዉን ሃሳብ የማየት እድል ያላቸዉ የምርምሩ ቡድን አባላት ብቻ ናቸዉ፡፡ጥናቱ በአንተ/አንቺ ላይ ምንም ዓይነት ጉዳት አያመጣም፡፡ ጥናቱን በማንኛዉም ሰዓት ማቋረጥ ትችላለህ፡፡

**የስምምነት ዉል**

ከላይ የተጠቀሰዉ የምርምር አላማ በባሌ ዞን በሚገኙ የከተማ መስተዳደሮች በሚገኙ ህብረተሰቦች መካከል ስለ ስኳር በሽታ ያላቸዉን የእዉቀት፣የአመለካከት እና የተግባር ደረጃ ለማወቅ እና እነዚህን የሚወስኑ ነገሮችን ለመለየት መሆኑ፤ በጥናቱ ለመሳተፍ በሙሉ ፈቃደኝነት ላይ የተመሰረተ መሆኑን፣የሰጠሁት ሃሳብ ሚስጥራዊነቱ የተጠበቀ፣በእኔ ላይ ምንም ዓይነት ጉዳት እደማይደርስብኝ ተነግሮኝና ተረድቼ በጥናቱ ተሳትፊያለሁ፡፡

እንደ ጥናቱ ተሳታፊ አካል ስለ ጥናቱ ወይም ስለ መብቴ ጥያቄ ቢኖረኝ ዋና ተመራማሪዉን; ጫንያለዉ ወርቁን ማነጋገር እንደምችል ገብቶኝ ተስማምቻለሁ፡፡

**የዋና ተመራማሪዉ አደራሻ:**

ስም:ጫንያለዉ ወረቁ ካሳሁን

ሞባይል ቁጥር: +251 921252375

 ኢሜል: chanyalewworku@gmail.com

**የመልስ ሰጪዉ ፊርማ________________ቀን _____________________**

**መጠይቅ የተጀመረበት ቀን : ________ሰዓት: _______ያለቀበት ሰዓት: _________.**

**የሱፐርቫይዘሩ ስም ________________________ፊርማ _________**

**ትዕዛዝ:** እባክዎ መላሹ የሚሰጠዉን መልስ ከጥያቄዎች ፊት ለፊት ካሉት አማራጮቹ መካከል ያክብቡ ወይም መላሹ የሚገልፀዉን ሀሳብ በቀኝ በኩል ባለዉ ክፍት ቦታ ላይ ይፃፉ፡፡

| **ክፍል-አንድ: የጥናቱ ተሳታፊዎች ማህበራዊ ኩነታት መረጃዎች** | | | | | | | | |  |  |
| --- | --- | --- | --- | --- | --- | --- | --- | --- | --- | --- |
| **ተ.ቁ** | | **ጥያቄ** | **የመልስ አማራጮች** | | | | **ምርመራ** | |  |  |
| 100 | | ጾታ | **0** = ወንድ  **1** = ሴት | | | |  | |  |  |
| 101 | | ዕድሜ | ________ዓመት | | | |  | |  |  |
| 102 | | የጋብቻ ሁኔታ | **0** = ያላገባ  **1** = ያገባ  **2** = የፈታ/የፈታች/ለየብቻ የምኖሩ  **3** = የሞተባት/የሞተችበት | | | |  | |  |  |
| 103 | | የትምህርት ደረጃ | **0** = ማንበብናመፃፍ የማይችል  **1** = ማንበብናመፃፍ የሚችል  **2** = ክፍል 1-4  **3** = ክፍል 5-8  **4** = ክፍል 9-12  **5** = ኮሌጅ እና ከዚያ በላይ | | | |  | |  |  |
| 104 | | የስራ ሁኔታ | 0= የቤት እመቤት  1 = ተማሪ  2 = ነጋዴ  3 = አርሶ አደር/አርብቶ አደር  4 = የመንግስት/የግል ተቀጣሪ  5 = የቀን ሰራተኛ  6 = ሌላ(ይገለፅ)_____________ | | | |  | |  |  |
| 105 | | አማካይ የቤተሰብ ወርሃዊ ገቢ | _______________ብር ( ኢትዮጵያ ብር) | | | |  | |  |  |
| 106 | | ስለስኳር በሽታ ሰምተዉ ያዉቃሉ? | 0 = አዎ  1 = የለም | | | |  | |  |  |
| 107 | | ስለስኳር በሽታ የጤና ትምህርት አገኚተዉ ያዉቃሉ | 0 = አዎ  1 = የለም | | | |  | |  |  |
| 108 | | ቴሌቪዥን/ራዲዮ አለዎት | 0 = አዎ  1 = የለም | | | |  | |  |  |
| 109 | | በቤተሰብ ዉስጥ የስኳር በሽታ የተያዘ አለ | 0 = አዎ  1 = የለም  2 = አላዉቅም | | | |  | |  |  |
| 110 | | ለያቄ ቁጥር -109 መልስዎ “አዎ” ከሆነ መረጃዉን ያገኙት ከየት ነዉ? | **0** = መገናኛ ብዙሃን  **1** = የጤና ባለሞያ  **2** = ጓደኛ/ዘመድ  **3** = ሌላ (መምህር, የሀይማኖት አባት…) | | | |  | |  |  |
| **ክፍል-ሁለት: የስኳር በሽታ እዉቀትን የሚመለከቱ ጥያቄዎች** | | | | | | | | | | |
| **ተ.ቁ** | | **ጥያቄዎች** | | | **የመልስ አማራጮች** | | | | | |
|  |  |  |  |  | **አዎ =1** | **አይደለም**  **= 2** | **አላዉቅም= 3** | | **ምርመራ** | |
|  | | **የስኳር በሽታ ማለት ምን ማለት ነው ?** | | | **1** | **2** | **3** | |  | |
| 200 | | የስኳር በሽታ ማለት ከቆሽት የሚመነጨው ኢንሱሊን የተባለው ሆርሞን (ንጥረ ነገር) ጭራሽ መጥፋቱ/አለመመንጨቱ ወይም መጠኑ መቀነሱ | | | **1** | **2** | **3** | |  | |
| 201 | | የስኳር በሽታ ማለት ከቆሽት የሚመነጨው ኢንሱሊን የተባለው ሆርሞን (ንጥረ ነገር) የሚያከናውነው ሥራ ሲሰናከል የሚመጣ ነዉ። | | | **1** | **2** | **3** | |  | |
| 202 | | የስኳር በሽታ ማለት ስኳር ወይም ጉሉኮስ በደም ውስጥ ከመጠን በላይ ሆኖ ሲገኝ የሚከሰት ነው፡፡ | | | **1** | **2** | **3** | |  | |
| 203 | | የስኳር በሽታ መዳን የማይችል ነዉ፡፡ | | | **1** | **2** | **3** | |  | |
| 204 | | የስኳር በሽታ ሁሉንም የሰዉነት አካል የሚያጠቃ ነዉ | | | **1** | **2** | **3** | |  | |
|  | | **ለስኳር በሽታ መከሰት ምክንያት ሊሆኑ የሚችሉ ጠንቆች አሉ?** | | |  |  |  | |  | |
| 205 | | የዕድሜ መጨመር (በእድሜ መግፋት) | | | **1** | **2** | **3** | |  | |
| 206 | | ዘር ወይም በቤተሰብ ውስጥ ሕመሙ ቀደም ብሎ መታየቱ | | | **1** | **2** | **3** | |  | |
| 207 | | ዉፍረት(ውፋሬ) ወይም ክብደት ከሚገባ በላይ መጨመር | | | **1** | **2** | **3** | |  | |
| 208 | | በርግዝና ወቅት በስኳር በሽታ መታመም | | | **1** | **2** | **3** | |  | |
| 209 | | የልተስተካከል የኑሮ ሁኔታ/የአመጋገብ ሁኔታ | | | **1** | **2** | **3** | |  | |
| 210 | | በቂ እንቅስቃሴ አለማግኘት | | | **1** | **2** | **3** | |  | |
|  | | **የስኳር በሽታ ምልክቶች ምንድን ናቸው?** | | |  |  |  | |  | |
| 211 | | አዘውትሮ /ቶሎ ቶሎ መሽናት | | | **1** | **2** | **3** | |  | |
| 212 | | በብዛት ውሃ መጠጣት | | | **1** | **2** | **3** | |  | |
| 213 | | በብዛት መራብ | | | **1** | **2** | **3** | |  | |
| 214 | | በብዛት ክብደት መቀነስ (መክሳት) | | | **1** | **2** | **3** | |  | |
| 215 | | በደም ዉስጥ ያለዉ ስኳር መጠን በብዛት መጨመር | | | **1** | **2** | **3** | |  | |
| 216 | | የዓይን ግርዶሽ | | | **1** | **2** | **3** | |  | |
| 217 | | በሰዉነት ላይ ያሉ ቁስሎች ቶሎ አለመዳን/ከቁስል በቶሎ ለመዳን አለመቻል | | | **1** | **2** | **3** | |  | |
| 218 | | የድካም ስሜት | | | **1** | **2** | **3** | |  | |
|  | | **የስኳር በሽታንና ጠንቆቹን እንዴት መከላከል ወይም እንዳይባባሱ ማድረግ ይቻላል?** | | |  |  |  | |  | |
| 218 | | በመርፌ መልክ በሚወሰዱ የተለያዩ የኢንሱሊን ዝግጅቶች የስኳር በሽታን መቆጣጠር ይቻላል | | | **1** | **2** | **3** | |  | |
| 219 | | በአፍ በሚወሰዱ በክኒን እና በጥቅል መልክ ባሉ መድሀኒቶች የስኳር በሽታን መቆጣጠር ይቻላል | | | **1** | **2** | **3** | |  | |
| 220 | | አዘውትሮ የሰውነት እንቅስቃሴ (ስፖርት) መሥራት። ለምሳሌ ያህል በየቀኑ ግማሽ ስዓት በመውሰድ በሳምንት ውስጥ ለአምስት ቀናት ያህል መሮጥ | | | **1** | **2** | **3** | |  | |
| 221 | | ጤነኛ የሆነ ምግብ መመገብ(ይህ ምግብ አትክልትና ፍራፍሬ በብዛት የያዘ ሆኖ) | | | **1** | **2** | **3** | |  | |
| 222 | | የዓይንን ጤንነት በሕክምና አዋቂ መመርመርና መንከባከብ | | | **1** | **2** | **3** | |  | |
| 223 | | የእግርን ጤንነት በሕክምና አዋቂ መመርመርና መንከባከብ | | | **1** | **2** | **3** | |  | |
| 224 | | ተገቢ በሆነ የሰውነት ክብደት ወይም ውፋሬ ላይ መገኘት | | | **1** | **2** | **3** | |  | |
|  | | **የስኳር በሽታ ጥንቃቄ ሳይደረግለት እንዲቀጥል ከተደረገ ምን ችግሮችን ያስከትላል?** | | |  |  |  | |  | |
| 225 | | የአይን በሽታን ብሎም መታወርን ማስከተል | | | **1** | **2** | **3** | |  | |
| 226 | | የኩላሊት በሽታን ማምጣት ወይም ማባባሰ | | | **1** | **2** | **3** | |  | |
| 227 | | የልብ በሽታ መከሰት | | | **1** | **2** | **3** | |  | |
| 228 | | በአንጎል ውስጥ ደም የመፍሰስ ወይም የመርጋት ችግር መፍጠር | | | **1** | **2** | **3** | |  | |
| 229 | | ለእግር መታመምና መቆረጥ ምክንያት መሆን | | | **1** | **2** | **3** | |  | |

| **ክፍል ሶስት: የጥናቱ ተሳታፊዎች ስለ ስኳር በሽታ ያላቸዉን አመለካከት የሚመለከቱ ጥያቄዎች** | | | | | | | | | | | | | |
| --- | --- | --- | --- | --- | --- | --- | --- | --- | --- | --- | --- | --- | --- |
| **ተ.ቁ** | **ጥያቄዎች** | | | **የመልስ አማራጮች** | | | | | | | | | |
|  |  |  |  | በጣም እስማማለሁ | | እስማማለሁ | | ገለልተኛ | | አልስማማም | | በጣም አልስማማም | ምርመራ |
| 300 | የስኳር በሽታ እዳለብኝ ሌሎች ቢያዉቁ እኔ አልረበሽም | | | **5** | | **4** | | **3** | | **2** | | **1** |  |
| 301 | ለስኳር በሽታ ምረመራ ማድረግ እንዳለብህ ታስባለህ | | | **5** | | **4** | | **3** | | **2** | | **1** |  |
| 302 | ለስኳር በሽታ የቤተሰብ አባሎችህ ምረመራ ማድረግ እንዳለባቸዉ ታስባለህ | | | **5** | | **4** | | **3** | | **2** | | **1** |  |
| 303 | ስለስኳር በሽታ ለመነጋገር የቤተሰብ እና የጓደኛ ድጋፍ ጠቀሜታ አለዉ ብለህ ታስባለህ | | | **5** | | **4** | | **3** | | **2** | | **1** |  |
| 304 | የስኳር በሽታን ለመቆጣጠር ብዙ ስኳር ከመመገብ መቆጠብ እዳለብህ አሰበህ ታዉቃለህ | | | **5** | | **4** | | **3** | | **2** | | **1** |  |
| 305 | የስኳር በሽታ በትዳር ዉስጥ ያለ ግንኙነትን ብዙም አይጎዳም | | | **5** | | **4** | | **3** | | **2** | | **1** |  |
| 306 | የስኳር በሽታ የእለት ከለት እንቅስቃሴን ያዉካል ብየ አላስብም | | | **5** | | **4** | | **3** | | **2** | | **1** |  |
| 307 | የአካል እንቅስቃሴ ማድረግ ለስኳር በሽታ ተጋላጭንትን ይቀንሳል ብለህ ታስባለህ | | | **5** | | **4** | | **3** | | **2** | | **1** |  |
| 308 | ሲጋራ ማጨስ ማቆምን ከጤና ባለሙ ጋር ተወያይተህ ተዉቃለህ | | | **5** | | **4** | | **3** | | **2** | | **1** |  |
| 309 | የተስተካከል የሰዉነት ክብደትን ጠብቆ መያዝ የስኳር በሽታን ለመቆጣጠር ይጠቅማል ብለህ ታስባለህ | | | **5** | | **4** | | **3** | | **2** | | **1** |  |
| 310 | በደም ዉስጥ ያለዉን የስኳር መጠን መቆጣጠር በስኳር በሽታ የሚመጡ ችገሮችን ሊከላከል ይችላል | | | **5** | | **4** | | **3** | | **2** | | **1** |  |
|  |  | | |  | |  | |  | |  | |  |  |
| **ክፍል አራት: ተሳታፊዎች ስኳር በሽታን ለመከላከል የሚያደርጉት ጥንቃቄንየሚመለከቱ ጥያቄዎች** | | | | | | | | | | | | | |
| **ተ.ቁ** | | **ጥያቄዎች** | **የመልስ አማራጮች** | | | | | | | | | | |
|  |  |  | በጣም ብዙ ጊዜ  = 5 | | ብዙ ጊዜ = 4 | | እርግጠኛ አይደለሁም =3 | | በጣም ትንሽጊዜ = 2 | | ምንም አላደርግምl =1 | | ምርመራ |
| 400 | | ቅባት ነክ የሆኑ ነገሮች የበዛበት ምግብ ትመገባልህ? |  | |  | |  | |  | |  | |  |
| 401 | | ከ 30-60 ደቂቃ የሚሆን የሰውነት እንቅስቃሴ (ስፖርት) በየቀኑ ትሰራልህ? ለምሳሌ ያህል በየቀኑ ግማሽ ስዓት ርምጃ ማደረግ፣የቤት ስራዎችን መስራት፣አቀበት መዉጣት…. | **5** | | **4** | | **3** | | **2** | | **1** | |  |
| 402 | | ጤናማ ክብደትህን እንዴት መጠበቅ እንዳለብህ የጤና ተሳትፎ አለህ? | **5** | | **4** | | **3** | | **2** | | **1** | |  |
| 403 | | አልኮል መጠጦችን ትጠጣለህ? ሲጋራስ ታጨሳለህ? | **5** | | **4** | | **3** | | **2** | | **1** | |  |
| 404 | | አዘዉትረህ የደም ግፊት መጠን ትከታተላለህ? | **5** | | **4** | | **3** | | **2** | | **1** | |  |

**ጨርሰናል! በጣም አመሰግናልሁ!!!!**

**S3 Table: Afan oroma version of the questionnaire**

**Gaafii Codi Lako .__________**

Qorannon kuun kan qophaa’e baraa 2008 a.l.h Godiina Baleeti bulchiiinsa magaalota godina magaleen Balee keesatti kan argaman keesatti dhibee sukaraa ilalchiisee bekuumisa,ilaalchafii sadaarkaa raawii kaneen kana waraa murteesan maalii akka ta’e ilaaluuf.

**Ibissa qorataa:** Maqaa : Caaniyaalaw worquu kaasaahun, Lak mobayilii : +251 921252375

**Hajaja : Filanno armangadii keessa deebii sirri jeettuuti mari**

| **Kuta-Tokko: Gafii Alala Qoratamtotaa Ilalu** | | | | | | | | |  |  |
| --- | --- | --- | --- | --- | --- | --- | --- | --- | --- | --- |
| **Lakka.** | | **Gafii** | **Filano** | | | | **Qoranno** | |  |  |
| 100 | | Saala | 0 = Dhiira 2 = Dhaala | | | |  | |  |  |
| 101 | | Umriin kee meeqa? | _____________ | | | |  | |  |  |
| 102 | | Haala Heerumaa/fuudha | 0 = Hin Heerumne/kan infuune  1= Heerumne/fuudhe  2 = Kan hiktee/hikee  3 = Najala du’e/duute | | | |  | |  |  |
| 103 | | Sadarkaa barumsaa | 0 = Baresuuf dubisuu hindanda’u  1= Dubisuuf baressuu kandanda’u  2 = Kutaa1-4  3 = Kutaa 5-8  4 = Kuuta 9-12  5 = Koleejiif sanaa oli | | | |  | |  |  |
| 104 | | Halla hojii | 0 = Hadha mana  1 = Barataa  2 = Daldalaa  3 = Qotee bulaa/horsise bulaa  4 = Qaxarii motuumaa/ dhunffaa  5 = Hojeeta guyaa  6= Kan biraa yojirate ibisii______ | | | |  | |  |  |
| 105 | | Galiin maatii jiii’an aviirajiidhan meeqa | _______________Qarshii | | | |  | |  |  |
| 106 | | Waa’ee dhibee sukaraaa dhagessanii bektuu | 0 = Eeyyee  1 = Lakki | | | |  | |  |  |
| 107 | | Waa’ee dhibee sukara barnoota argatanii beektuu | 0 = Eeyyee  1 = Lakki | | | |  | |  |  |
| 108 | | Teeleviiziinii / raadiiyoo qabduu | 0 = Eeyyee  1 = Lakki | | | |  | |  |  |
| 109 | | Maatii keesa dhibee sukaaran kan qabamee jiraa | 0 = Eeyyee  1 = Hin jiiru  2 = Hinbeeku | | | |  | |  |  |
| 110 | | Gaafii lakko. -109 debiin kessan “eeyyee taa’e” odeeffannoo kana eessaa argattan? | 0 = Media  1 = Ogeessa fayya irra  2 = Hiriyaa/ Firaa irra  3 = Kan biroo (barsiisaa,abbaaa hamantaairaa) | | | |  | |  |  |
| **Kuutaa Lamafaa: Gaafii Bekumssa Dhibee Sukkara Ilaalan** | | | | | | | | | | |
| **Lak.** | | **Gaffiwaan** | | | **Filanno Gafii** | | | | | |
|  |  |  |  |  | **Eeyyee=1** | **Lakki**  **= 2** | **Hinbeeku= 3** | | **Qoranno** | |
|  | | **Dhiibee Sukaaraa Jeechuun Maalii?** | | |  |  |  | |  | |
| 200 | | Dhiibee sukaara jechuu rajiijin yeeroo hormoonii insulin jedhaamu burqisisu dhisuu dha. | | | **1** | **2** | **3** | |  | |
| 201 | | Dhiibee sukkaara jechuu insuliinin rajii jirra maddu yeroo hojii isaa dehaabudha ykn dangamudia. | | | **1** | **2** | **3** | |  | |
| 202 | | Dhiibee sukkaara jechuun sukaarri yokeen gulukosiin garmalee dhiga keesatti yeroobayatee argamuu kan uumamuu dha. | | | **1** | **2** | **3** | |  | |
| 203 | | Dhiibeen sukkaaraa fayyuu kan hindandeenyeedha. | | | **1** | **2** | **3** | |  | |
| 204 | | Dhibeen suukaraa qaaama namaa hunda kan midhudha. | | | **1** | **2** | **3** | |  | |
|  | | **Humama Dhibee Sukarattif Wantonii Sababa Ta’anii Nijiiruu ?** | | |  |  |  | |  | |
| 205 | | Uumirii dabaluu /duloomu | | | **1** | **2** | **3** | |  | |
| 206 | | Sanyii /maatii keessa dhibeen kun dursee mulachuu | | | **1** | **2** | **3** | |  | |
| 207 | | Furdina ykn ulfaatina gaamaa hamma barlaadamuu oli dabaluu | | | **1** | **2** | **3** | |  | |
| 208 | | Yeroo ulfaa/garatti batan dhibee sukaratiin qabamuu | | | **1** | **2** | **3** | |  | |
| 209 | | Jireenya mijawaa hintaane / nyaata mijawaa hintaane | | | **1** | **2** | **3** | |  | |
| 210 | | Sochii qaama ga’a ta’e dhabuu | | | **1** | **2** | **3** | |  | |
|  | | **Malattoleen Dhibee Sukaraa Ta’aan Kamiidha** | | |  |  |  | |  | |
| 211 | | Yeroo hunda /dafanii dafanii fincaa’uu | | | **1** | **2** | **3** | |  | |
| 212 | | Baayiinaan bishaan dhuguu | | | **1** | **2** | **3** | |  | |
| 213 | | Bayiisanii belaa’u | | | **1** | **2** | **3** | |  | |
| 214 | | Ulfiin qaama baayyee hiracuu/huqaacuu | | | **1** | **2** | **3** | |  | |
| 215 | | Bayiin sukaaraa qamaa keesa jiruu bayiisee dabaluu | | | **1** | **2** | **3** | |  | |
| 216 | | Ijji ilaaluuf rakkachuu | | | **1** | **2** | **3** | |  | |
| 217 | | Madaan qaama irra jiru dafee fayuu diduu/madairaa dafaniifayuu dhabuu | | | **1** | **2** | **3** | |  | |
| 218 | | Dadhabiin dhagaamuu. | | | **1** | **2** | **3** | |  | |
|  | | **Dhiibee Sukaarafii Sababa Ka’umisa Isaa Akkamitti Dhoowuufii Akka Hin Hammannee Gocuun Danda’ma?** | | |  |  |  | |  | |
| 218 | | Haala marfeetiin qoriicha fudhatamaan gosaawan insuulinitiin dhibee sukaara too’achuun nidanda’amaa. | | | **1** | **2** | **3** | |  | |
| 219 | | Qoorsota haala kininitin fi maramani afanniin fudhatamanii fi dhukkuba sukkaarra too’achuun ni danda’amaa | | | **1** | **2** | **3** | |  | |
| 220 | | Sochi qaamaa walittifufinsa fabu hojiachuu,fakkeemaaf quyyaatti sa’aatin walkkaa fudhachuun torbanitti quyyoota shanif kaachuu | | | **1** | **2** | **3** | |  | |
| 221 | | Nyaata fayyaa qabeerssa ta’an nyaachuu (fkn muduraafi fuduraa kanta’an) | | | **1** | **2** | **3** | |  | |
| 222 | | Fayaa ijaa ogeessa fayaatiin yaaalamuufi ilaalamu/kunuunfamuu | | | **1** | **2** | **3** | |  | |
| 223 | | Fayyaa miilaa ogeessa faatiin ilalaamuufi yalamuuu | | | **1** | **2** | **3** | |  | |
| 224 | | Ulfeenyaaf furdiina madalaawa ta’ee irratti argamuu | | | **1** | **2** | **3** | |  | |
|  | | **Offi Eggannoo Dhibeee Sukaara Osoo Hin Tasiisin Yooturamee Rakoo Maaltuu Humamaa?** | | |  |  |  | |  | |
| 225 | | Dhibee ijaa fiduu tureesi jaamina ijaa fiduu | | | **1** | **2** | **3** | |  | |
| 226 | | Dhibee kalee fiduufi ykn kakaasuu | | | **1** | **2** | **3** | |  | |
| 227 | | Dhibee dnnee namatti fiduu | | | **1** | **2** | **3** | |  | |
| 228 | | Rakkoo sammuu kessatti dhiiginii dhangala’uu ykn dhiiginii itituutu uumuu | | | **1** | **2** | **3** | |  | |
| 229 | | Dhibee lukka fiduufi hamma muramuutti geessuuf sababa ta’uu | | | **1** | **2** | **3** | |  | |

| **Kutaa Sadaffaa : Ilaalich Dhibee Sukaaratiif Qbaan Kan Ilaalu Dha.** | | | | | | | | | | | | |
| --- | --- | --- | --- | --- | --- | --- | --- | --- | --- | --- | --- | --- |
| **Lak.** | **Gaafiwaan** | | **Filannoo Gaafii** | | | | | | | | | |
|  |  |  | Sirritan fudhadha t | | Nan fudhadha | | Yaada hinqabuu | | Hin fudhadhu | Siriiti hinfudhadhu | | Qoranna |
| 300 | Dhibbeen sukaara aka nara jiruu namooni biraa yoobeekani anii hin jeeqamu | | **5** | | **4** | | **3** | | **2** | **1** | |  |
| 301 | Dhibee sukaaraatiif qorannoo gochuun narajiraa jettee niyaada. | | **5** | | **4** | | **3** | | **2** | **1** | |  |
| 302 | Dhibee sukaaratiif maatiinkee qorannoo gochuu qaabuu jeettee niyaada. | | **5** | | **4** | | **3** | | **2** | **1** | |  |
| 303 | Waa’ee dhiibee suukaraa maatiifi hiriiyaaa faana hasaawhun gargaarsaafii faayiida qabaa jeettee yaadda. | | **5** | | **4** | | **3** | | **2** | **1** | |  |
| 304 | Dhibee sukaaraa irraa ofii dhoorkuuf nyaata sukaara qabuu hirisuun fayiida qaabaa jette yadee beekitta? | | **5** | | **4** | | **3** | | **2** | **1** | |  |
| 305 | Dhibeen suukaraa hariiroo dhirsaafi nitii giduuti rakoo humaa hinfiidu | | **5** | | **4** | | **3** | | **2** | **1** | |  |
| 306 | Dhibeen sukkaraa sochi quyyaa dhaa quyyati taasaisu keersatti nakko fida jedhee hinyaadu | | **5** | | **4** | | **3** | | **2** | **1** | |  |
| 307 | Sochii qaamaa gochuun conraa dhukkuba sukkaraatif saaxilamuu nihri isa jeffeeyaaddaa | | **5** | | **4** | | **3** | | **2** | **1** | |  |
| 308 | Sijaaraa xuuxu dhabuun garee ogeeyii faayaa wajiin mariyaattee beektaa? | | **5** | | **4** | | **3** | | **2** | **1** | |  |
| 309 | Hulfiina qamaa sirrita’e tursiisani eeguun dhibee sukaara to’achuuf nifayaada jeetee niyaada? | | **5** | | **4** | | **3** | | **2** | **1** | |  |
| 310 | Baayiina suukaraa dhigaa kessa jiru too’achuun Rakkoo sabaaba dhibee sukaaratiin dhufuu dhorkuun idandaa’ama. | | **5** | | **4** | | **3** | | **2** | **1** | |  |
| **Kuutaa Afraffa : Hirmattoni Dhibee Sukaraa Dhorkuuf Offi Eganno Godhaan Kanilaaludha** | | | | | | | | | | | | |
| **Lak.** | **Gafiiwan** | **Filanno deebii** | | | | | | | | | | |
|  |  | Yeroo baayee  = 5 | | Yeroo xiqoo = 4 | | hinbeeku=3 | | Yeroobayee xiqqoo= 2 | | | Humaa hingoodhu =1 | qoranno |
| 400 | Nyaata qiibatii bayaatu ni soratta? | **5** | | **4** | | **3** | | **2** | | | **1** |  |
| 401 | Daqiiqa 30-60 kaniita’u sochii qaamaa (spoortii) nii hojeetaa fkn sa’a ti walakkaa tarkaanfii ,hojii manaa tabbaa ba’uu buu’u nitaasiftaa | **5** | | **4** | | **3** | | **2** | | | **1** |  |
| 402 | Hulfiina fayaaleesa ta’e akamiiti eguu akka dandeesu tatafii fayaa nigoota? | **5** | | **4** | | **3** | | **2** | | | **1** |  |
| 403 | Dhuugatii nama macheesan fii sigaara ni xuxaa? | **5** | | **4** | | **3** | | **2** | | | **1** |  |
| 404 | Yeroo mara dhiibbaa dhiiga keetii ni hordoofta? | **5** | | **4** | | **3** | | **2** | | | **1** |  |

**Fiixeraa ! Baayyee Isiin Galateefadha!!!!**
